# A Whole-Brain Connectivity Map of VTA and SNc Glutamatergic and GABAergic Neurons in Mice

**DOI:** 10.3389/fnana.2021.818242

**Published:** 2021-12-23

**Authors:** Sile An, Xiangning Li, Lei Deng, Peilin Zhao, Zhangheng Ding, Yutong Han, Yue Luo, Xin Liu, Anan Li, Qingming Luo, Zhao Feng, Hui Gong

**Affiliations:** ^1^Britton Chance Center for Biomedical Photonics, Wuhan National Laboratory for Optoelectronics, MoE Key Laboratory for Biomedical Photonics, Huazhong University of Science and Technology, Wuhan, China; ^2^Huazhong University of Science and Technology (HUST)-Suzhou Institute for Brainsmatics, Jiangsu Industrial Technology Research Institute (JITRI), Suzhou, China; ^3^School of Biomedical Engineering, Hainan University, Haikou, China

**Keywords:** ventral tegmental area (VTA), substantia nigra pars compacta (SNc), cell-type-specific, virus tracers, whole brain, three-dimension

## Abstract

The glutamatergic and GABAergic neurons in the ventral tegmental area (VTA) and substantia nigra pars compacta (SNc) mediated diverse brain functions. However, their whole-brain neural connectivity has not been comprehensively mapped. Here we used the virus tracers to characterize the whole-brain inputs and outputs of glutamatergic and GABAergic neurons in VTA and SNc. We found that these neurons received similar inputs from upstream brain regions, but some quantitative differences were also observed. Neocortex and dorsal striatum provided a greater share of input to VTA glutamatergic neurons. Periaqueductal gray and lateral hypothalamic area preferentially innervated VTA GABAergic neurons. Specifically, superior colliculus provided the largest input to SNc glutamatergic neurons. Compared to input patterns, the output patterns of glutamatergic and GABAergic neurons in the VTA and SNc showed significant preference to different brain regions. Our results laid the anatomical foundation for understanding the functions of cell-type-specific neurons in VTA and SNc.

## Introduction

The ventral tegmental area (VTA) and substantia nigra pars compacta (SNc), as nuclei associated with dopamine release, are involved in reward processing, reinforcement learning, and motor control (Wise, 2004; Fields et al., 2007; Ikemoto, 2007; Cohen et al., 2012). Dysfunction in these two brain regions can also lead to some neurological disorders such as drug addiction, depression and Parkinson’s disease (Wise, 2004; Nestler and Carlezon, 2006; Deisseroth, 2014; Lammel et al., 2014; Volkow and Morales, 2015; Luscher, 2016; Schultz, 2016). Many previous studies have focused on the neural connectivity of VTA and SNc dopaminergic neurons in mice (Watabe-Uchida et al., 2012; Ogawa et al., 2014; Beier et al., 2015; Lerner et al., 2015; Menegas et al., 2015). These works have contributed to understanding the role of dopaminergic neurons in the VTA and SNc related to physiological functions and neurological disorders.

However, VTA and SNc are heterogeneous nuclei in neurotransmitter types. These two brain regions also contained glutamatergic and GABAergic neurons (Yamaguchi et al., 2007, 2011; Nair-Roberts et al., 2008). These two types of neurons in the VTA and SNc also play pivotal roles in multiple brain functions such as sleep and wakefulness (Yu et al., 2019), innate defensive behaviors (Barbano et al., 2020), and graded locomotor movements (Ryczko et al., 2017). But their whole-brain input-output connectivity has not been comprehensively mapped and analyzed.

The neural connectivity of VTA and SNc has previously been investigated using classical anterograde or retrograde tracers (Geisler et al., 2007; Yetnikoff et al., 2014). Nonetheless, classical tracing methods lack cell-type specificity and therefore, they are not capable of identifying the whole-brain input and output connections of a certain genetically well-defined neuronal population. With recent advances in both virus-assisted circuit tracing and whole-brain three-dimensional optical imaging techniques, it is now available to map cell-type-specific of neural connectivity in specific brain regions (Wickersham et al., 2007; Wall et al., 2010; Huang and Zeng, 2013; Callaway and Luo, 2015). Recent studies have used these techniques to characterize inputs of VTA or SNc dopaminergic neurons (Watabe-Uchida et al., 2012; Ogawa et al., 2014; Beier et al., 2015; Lerner et al., 2015; Menegas et al., 2015). Faget et al. (2016) have used these techniques to identify afferents to different neuron types in the VTA. These studies have greatly enhanced our understanding of the input circuits of cell-type-specific neurons in the VTA and SNc. However, these works did not investigate the output circuits of cell-type-specific neurons in the VTA and SNc. Specially, there is less work to study the neural circuits of cell-type-specific neurons in the SNc using the virus-assisted circuit tracing technique.

Here, we used a modified rabies virus (RV) and adeno-associated virus (AAV) in combination with the Cre/LoxP system to map and compare the whole-brain inputs and outputs of glutamatergic and GABAergic neurons in the VTA and SNc. We provided a whole-brain analysis of bidirectional connectivity to glutamatergic and GABAergic neurons in the VTA and SNc. The input patterns to different cell types in the VTA and SNc were similar, with dominant and comparable projections from the periaqueductal gray, superior colliculus, lateral hypothalamic area and striatum. But some quantitative differences were also observed, with neocortex and dorsal striatum providing a greater share of input to VTA glutamatergic neurons, periaqueductal gray and lateral hypothalamic area preferentially innervating VTA GABAergic neurons. SNc GABAergic neurons received proportionally more inputs from the ventral striatum. Specially, superior colliculus provided more inputs to SNc glutamatergic neurons compared to GABAergic neurons. Compared to input patterns, the output patterns to glutamatergic and GABAergic neurons in the VTA and SNc showed remarkable differences in different brain regions. These results provide neural connectivity information for further revealing the functional relevance of VTA and SNc.

## Materials and Methods

### Animals

All of the animal experiments were approved by the Animal Ethics Committee of Huazhong University of Science and Technology. Adult (2–4 months old) VGLUT2-cre mice and VGAT/GAD2-Cre mice were used in this study, targeting glutamatergic neurons and GABAergic neurons, respectively. All mice were housed under a 12/12 h light/dark cycle at 25°C, and food and water were available *ad libitum*.

### Virus Information

All the viral vectors were provided by BrainVTA (BrainVTA Co., Ltd., Wuhan, China). For the transsynaptic retrograde tracing experiments, two adeno-associated viruses (AAV) as helpers and one modified rabies virus (RV) were used. AAV-DIO-TVA-BFP (2 × 10^12^ viral genomes/ml) and AAV-DIO-RG (2 × 10^12^ viral genomes/ml) were mixed at a 1:2 ratio of viral particles. For the output tracing experiments, AAV-DIO-EYFP (2 × 10^12^ viral genomes/ml) was used.

### Animal Surgery and Viral Injections

Before viral injection, the mouse was anesthetized with pentobarbital sodium and mounted in a stereotaxic holder. For the transsynaptic retrograde tracing experiments, 150 nl of helper AAV were stereotactically injected into the VTA (bregma −3.28 mm, lateral 0.5 mm, depth 5.0 mm, according to Allen Reference Atlas) (*n* = 10 mice) or the SNc (bregma −3.18 mm, lateral 1.5 mm, depth 4.75 mm, according to Allen Reference Atlas) (*n* = 10 mice) using a pressure injection pump (Nanoject II: Drummond Scientific, Co., Broomall, PA, United States). Then the mouse was placed in a clean cage for recovery and AAV expression. After 3 weeks, 200 nl of RV-EnvA-DG-GFP was injected into the same coordinates with the same procedure mentioned above. All rabies virus tracing experiments were completed in Biosafety level 2 (BSL2) Laboratory. For axonal projections experiments, 50 nl AAV-DIO-EYFP was injected into the VTA (bregma −3.28 mm, lateral 0.5 mm, depth 5.0 mm, according to Allen Reference Atlas) (*n* = 9 mice) or the SNc (bregma −3.18 mm, lateral 1.5 mm, depth 4.75 mm, according to Allen Reference Atlas) (*n* = 9 mice) following the same procedures above. After 28 days, these mice were perfused.

### Histology

All histological operations followed previous studies (Gong et al., 2016; Ren et al., 2018). One week after the injection of rabies virus, mice were anesthetized and transcardially perfused with 0.01 M phosphate-buffered saline (PBS, Sigma-Aldrich, United States) followed by 4% paraformaldehyde (PFA, Sigma-Aldrich, United States) in 0.01 M PBS. Mouse brains were removed and then post-fixed in 4% PFA solution for 48 h. To acquire the three-dimensional mouse brain dataset, the brains were embedded with a graded glycol methacrylate (GMA, Ted Pella, Inc., Redding, CA, United States) for the fluorescence micro-optical sectioning tomography (fMOST).

### Microscopy Imaging and Analysis

Whole-brain sections were imaged with the fMOST (Gong et al., 2016). Briefly, the embedded sample was fixed on a high-precision 3D translation stage (ABL20020-ANT130-AVL125, Aerotech Inc.). Then the image of the top surface acquired with two simultaneous fluorescent channels, the imaged tissue was subsequently removed via a diamond knife (Diatome AG). Finally, we could obtain continuous whole brain dataset layer by layer at a voxel resolution of 0.32 μm × 0.32 μm × 2 μm. For starter cell location and counting, we collected 70 μm local sections near the injection site from 12 brains and every second slice was imaged with confocal microscopy (Leica SP8). For the dual-color RV imaging, the coronal sections were mounted with 50% glycerol (vol/vol) and imaged using a × 10, 0.45 NA objective (Olympus versus 120 virtual microscopy slide scanning system, Olympus).

For starter cell counting, the starter cells were manually counted using the Cell Counter ImageJ plug-in. For whole-brain input neuron location and counting, the input neurons were located and quantified automatically using Neuron Global Position System (NeuroGPS) (Quan et al., 2013) and manually checked and corrected some indiscernible mistake results. Then spatial information of the neurons was registered into the Allen Mouse Brain Common Coordinate Framework (CCFv3) to facilitate 3D whole-brain quantification analysis and visualization.

For output tracing analysis, we first registered down sampling coronal sections (2 μm × 2 μm × 2 μm) to the CCFv3. The registration method used has been previously described (Ni et al., 2020). Then we applied the modified maximum entropy threshold segmentation algorithm to registered coronal images to segment fluorescence fiber signals from background. In order to ensure the accuracy of segmentation, we manually checked and corrected the segmentation results. Next, we calculated the signal density of binary images in each brain region of interest. In each segmented image, signal density was calculated in the following manner: using the sum of detected pixels in each brain region of interest divided by sum of whole brain fluorescence signals pixels. Then the signal density matrix was used for further quantification analysis and 3D whole-brain visualization in the Amira software (v6.1.1, FEI).

### Statistical Analysis

All statistical graphs were generated using Graphpad Prism v.8.0.2 and Microsoft Excel (Office 2016). To quantify the similarity about input and output patterns, we calculated Pearson’s correlation coefficients. Then the correlation coefficients were hierarchically clustered using R language. For statistical analysis, one-way ANOVA followed by the Tukey’s *post hoc* tests or the Bonferroni correction were performed using SPSS (version 24.0). All of the results are presented as the mean ± standard error of the mean (SEM).

## Results

### Viral Tracing Strategy to Identify Whole-Brain Inputs and Outputs of Glutamatergic and GABAergic Neurons in the VTA and SNc

**FIGURE 1:**
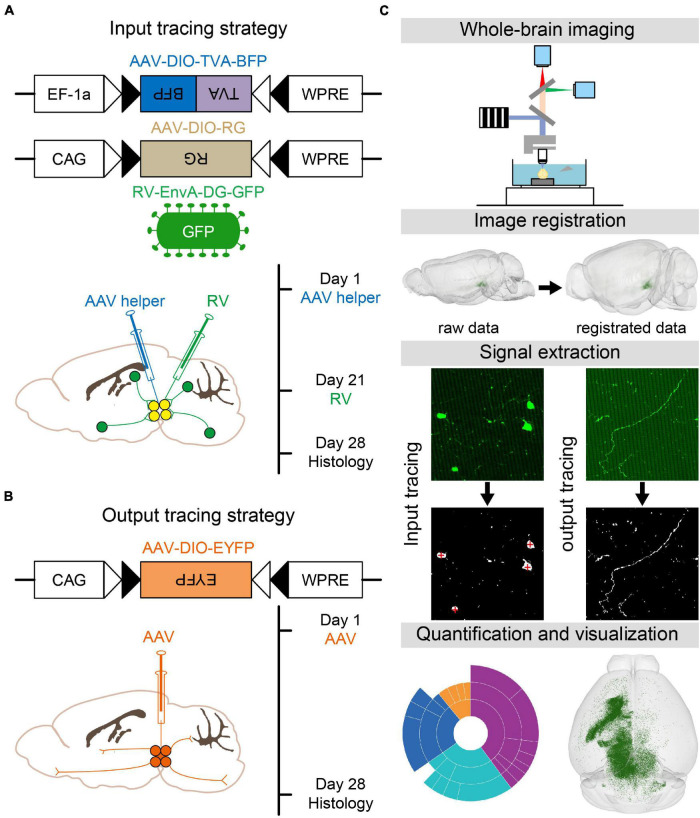
Identification of whole-brain inputs and outputs of glutamatergic and GABAergic neurons in VTA and SNc. **(A)** Design of viral vectors and injection procedure for RV-mediated transsynaptic retrograde tracing. **(B)** Design of viral vector and injection procedure for tracing VTA and SNc output projections. **(C)** Main steps for data generation and processing.

To map the whole-brain monosynaptic inputs to glutamatergic and GABAergic neurons in VTA and SNc, we used RV-mediated transsynaptic retrograde tracing, which used the modified rabies virus EnvA-DG-GFP combined with a Cre/LoxP gene-expression strategy (Wickersham et al., 2007; Miyamichi et al., 2011; Watabe-Uchida et al., 2012; Wall et al., 2013). First, two AAV helper viruses (AAV-DIO-TVA-BFP and AAV-DIO-RG) were injected unilaterally into the VTA or SNc in VGLUT2-Cre or VGAT/GAD2-Cre mice, respectively. After 3 weeks of the expression of AAV helper viruses, RV-EnvA-DG-GFP was injected into the same position. One week after the injection of rabies virus, the mice were perfused and further processed for imaging (Figure 1A).

To map the whole-brain outputs of glutamatergic and GABAergic neurons in the VTA and SNc, we stereotaxically injected Cre-dependent AAV virus (AAV-DIO-EYFP) into the VTA or SNc in VGLUT2-Cre or VGAT/GAD2-Cre mice. After 28 days, the brain tissues were perfused (Figure 1B).

After the brain tissues were perfused, the brain samples were automatically and continuously imaged over 5000 coronal whole-brain sections per mouse using the fMOST, with the voxel resolution of 0.32 μm × 0.32 μm × 2 μm (Gong et al., 2016). Then the dataset was registered to the CCFv3 to facilitate 3D whole-brain quantification analysis and visualization (Figure 1C; Wang et al., 2020).

To verify whether the injection site of input tracing sample was correct before further analysis, we co-expressed the avian receptor TVA and rabies glycoprotein G (RG) for each cell type by injecting two AAV helper viruses (AAV-DIO-TVA-BFP and AAV-DIO-RG) into VGLUT2-Cre or GAD2-Cre mice. Injection sites were targeted to either the VTA or SNc. Three weeks later, RV-EnvA-DG-GFP was injected into the same area. This modified RV can only infect the cells expressing TVA and requires RG to spread retrogradely to presynaptic cells. One week after the injection of rabies virus, the mice were sectioned and imaged.

The co-expression of AAV-BFP and RV-GFP neurons were defined as starter cells as in previous studies (Watabe-Uchida et al., 2012). We observed massive green fluorescent protein (GFP)-labeled neurons (Supplementary Figures 1A,D) and the starter cells at the injection region (Supplementary Figures 1A,B). For each group, the location of the vast majority of starter cells was restricted to the VTA and SNc (Supplementary Figures 1A,C), although we found a small number of co-expression of AAV-BFP and RV-GFP neurons in neighboring nuclei: the midbrain reticular nucleus (MRN), substantia nigra, reticular part (SNr), midbrain reticular nucleus, retrorubral area (RR). However, these neurons made up a small fraction of total starter neurons in our experimental mice (Supplementary Figure 1C). Although the numbers of labeled neurons varied, there was a linear relationship between the number of starter cells and input cells (Supplementary Figure 1E). For output tracing, the accuracy of the injection site was confirmed by both the cytoarchitecture information provided by propidium iodide (PI)-staining and the soma morphology of cells near the injection site. We evaluated qualitatively the accuracy of the injection according to the coronal images near the injection sites. We found that the majority of cell bodies was located in the injection region and only few cell bodies were outside the injection region, for example, MRN (Supplementary Figure 1F). These results demonstrated that our tracing strategy was feasible for mapping the whole brain inputs and outputs of glutamatergic and GABAergic neurons in the VTA and SNc.

### Whole-Brain Monosynaptic Inputs of Glutamatergic and GABAergic Neurons in the VTA and SNc

**FIGURE 2:**
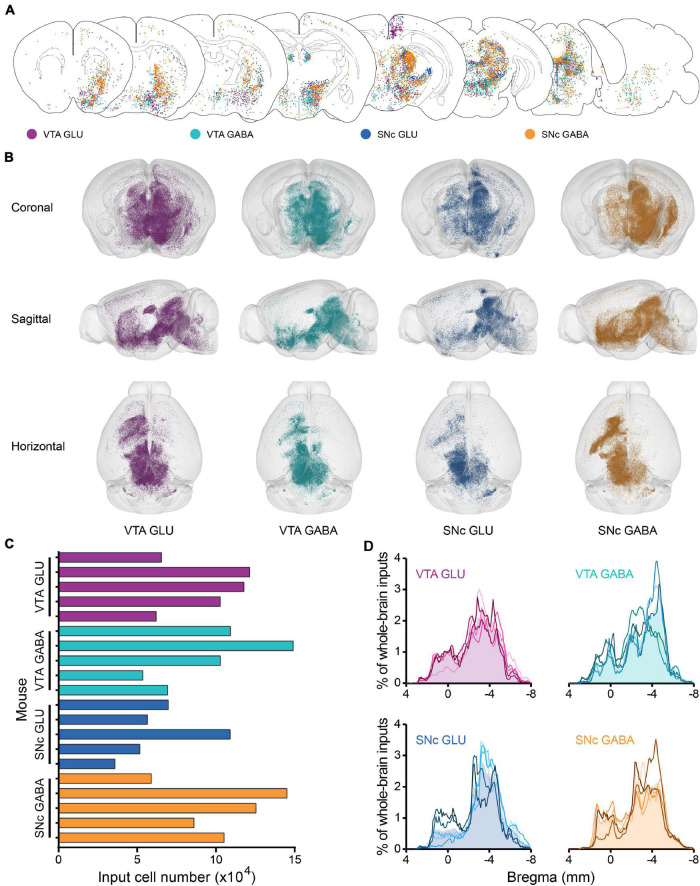
Whole-brain monosynaptic inputs to glutamatergic and GABAergic neurons in VTA and SNc. **(A)** Schematic coronal sections showing labeling of monosynaptic inputs to glutamatergic and GABAergic neurons in VTA and SNc (hereafter called VTA GLU, VTA GABA, SNc GLU, and SNc GABA neurons). One dot represents one neuron. **(B)** 3D visualization of whole-brain monosynaptic inputs to glutamatergic and GABAergic neurons in VTA and SNc from different view. **(C)** Numbers of transsynaptically labeled neurons (“input neurons”), each row represents the input neurons in each mouse. **(D)** Whole-brain distribution of all input neurons along the A-P axis. Colored lines, input distribution for the individual mouse; colored line with the shaded area under it, average input distribution.

To reveal the whole-brain distribution of GFP-labeled neurons more clearly, we performed the 50 μm maximum intensity projection on continuous 2 μm coronal images (Figure 2A and Supplementary Figure 2). We found that glutamatergic and GABAergic neurons in the VTA and SNc integrate inputs from discrete brain areas, including cortical plate (MOp, MOs, SS, AI, RSP, VIS), cortical subplate (BLA), striatum (ACB, CP), pallidum (BST), thalamus (LH), hypothalamus (MPO, LPO, TU, LHA, ZI), midbrain (MRN, VTA, IF, SNr, NOT, PAG, SNc, SC, APN, IPN, RR, CLI, DR), hindbrain (PRNc, GRN, IO, RPA). Overall, the GFP-labeled neurons were predominantly found ipsilateral to the injection site, although sparser labeling was also found in the contralateral hemisphere (Figure 2A and Supplementary Figure 2).

In order to observe more intuitively the whole-brain input distribution of glutamatergic and GABAergic neurons in VTA and SNc, we performed the three-dimensional visualization. Each input neuron was represented by a small globule in the 3D models (Figure 2B). Obviously, the whole-brain input distribution of glutamatergic and GABAergic neurons in the VTA and SNc was similar in most brain regions from the horizontal view (Figure 2B). Most of the input neurons were mainly concentrated in the forebrain and midbrain nuclei that are close to the injection site. But some differences were also observed in some brain regions from the sagittal view (Figure 2B), such as the striatum.

To compare the input patterns among four groups, first we quantify the whole-brain monosynaptic input distributions of glutamatergic and GABAergic neurons in the VTA and SNc. We counted the numbers of transsynaptically labeled neurons in each brain (*n* = 5 mice each for the VTA GLU, VTA GABA, SNc GLU, and SNc GABA groups) (Figure 2C). The numbers of input neurons among four groups were similar (Figure 2C). To correct for the variability in the total number of neurons, the number of the input neurons within each brain region was normalized by the total number of input neurons. Then we investigated the whole-brain distributions of all input neurons along the A-P axis (Figure 2D). The overall distribution pattern of the input neurons to glutamatergic and GABAergic neurons in the VTA and SNc were also similar among one another. Two peaks were formed at bregma 0 and −4 mm positions, as the results of previous qualitative observations. The input neurons were mainly concentrated in the forebrain such as the striatum and the midbrain that were close to the injection site.

### Quantitative Comparisons of Whole-Brain Monosynaptic Inputs of Glutamatergic and GABAergic Neurons in the VTA and SNc

**FIGURE 3:**
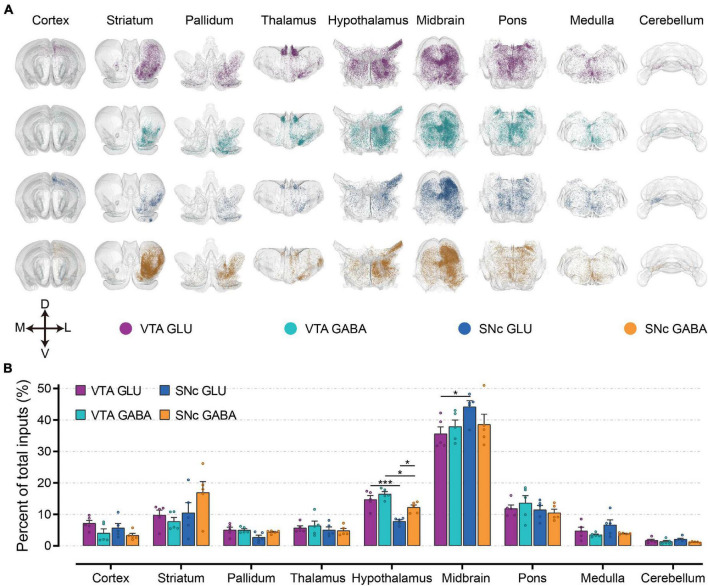
Whole-brain monosynaptic inputs to glutamatergic and GABAergic neurons in VTA and SNc in nine brain areas. **(A)** 3D visualization of whole-brain monosynaptic inputs to glutamatergic and GABAergic neurons in VTA and SNc in nine brain areas. M, medial; L, lateral; D, dorsal; V, ventral. **(B)** Proportions of total inputs from nine brain areas. Mean ± SEM (*n* = 5 mice each for the VTA GLU, VTA GABA, SNc GLU, and SNc GABA groups). ***p < 0.001 and *p < 0.05. Only significant differences between the same cell type in different brain areas or between cell types in the same brain areas are marked; one-way ANOVA followed by Tukey’s *post hoc* tests. The percentages of inputs in nine brain areas are listed in Supplementary Data 1.

To first gain an overview of the whole-brain monosynaptic input proportions of glutamatergic and GABAergic neurons in the VTA and SNc, we quantified the proportions of input neurons in nine grouped brain areas (Figures 3A,B). 3D visualization of whole-brain monosynaptic inputs to glutamatergic and GABAergic neurons in the VTA and SNc in these nine brain areas were performed according to the CCFv3 (Figure 3A). As expected, the midbrain (excluding the VTA/SNc) contributed most of the inputs to glutamatergic and GABAergic neurons in the VTA and SNc (Figure 3B). Compared to GABAergic neurons, glutamatergic neurons in the VTA and SNc received more inputs from cortex and medulla (Figure 3B). Striatum provided a greater share of input to SNc GABAergic neurons (Figure 3B). Compared to SNc, VTA glutamatergic and GABAergic neurons received proportionally more inputs from the pallidum and hypothalamus (Figure 3B). Glutamatergic and GABAergic neurons in VTA and SNc received similar inputs from thalamus and pons (Figure 3B). Cerebellum provided the least proportional inputs to glutamatergic and GABAergic neurons in VTA and SNc (Figure 3B).

**FIGURE 4:**
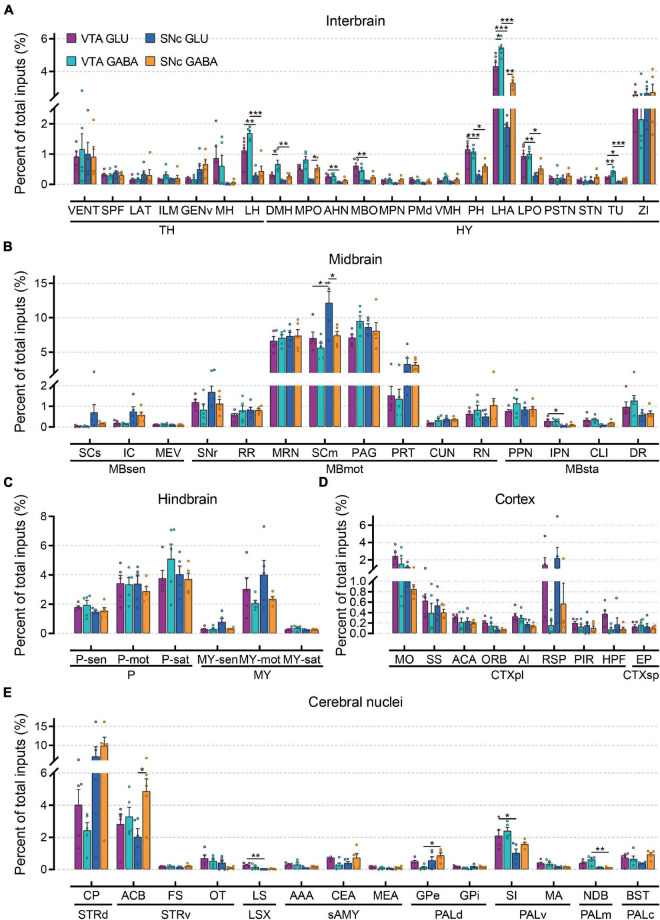
Quantitative comparisons of whole-brain input to glutamatergic and GABAergic neurons in VTA and SNc in individual brain regions. **(A)** Proportions of total inputs from interbrain region. **(B)** Proportions of total inputs from midbrain region. **(C)** Proportions of total inputs from hindbrain region. **(D)** Proportions of total inputs from cortex region. **(E)** Proportions of total inputs from cerebral nuclei region. Mean ± SEM (*n* = 5 mice each for the VTA GLU, VTA GABA, SNc GLU, and SNc GABA groups). ***p < 0.001, **p < 0.01, and *p < 0.05. Only significant differences between the same cell type in different brain regions or between cell types in the same brain regions are marked; one-way ANOVA followed by Tukey’s *post hoc* tests. Abbreviations of the 67 brain regions and their percentages of inputs are listed in Supplementary Table 1 **and** Data 1.

To compare monosynaptic inputs in more detail, we further quantified the input neurons in each of 67 individual brain nuclei (Figure 4 and Supplementary Figure 3). In the interbrain (Figure 4A): compared to thalamus, hypothalamus provided a higher proportion of input to glutamatergic and GABAergic neurons in VTA and SNc, especially for LHA and ZI. In addition, VTA glutamatergic and GABAergic neurons received more inputs from MH, LH, MPO, AHN, MBO, PH, LHA, LPO, and TU compared to SNc glutamatergic and GABAergic neurons. Within the VTA, the VENT, ILM, LH, DMH, MPO, VMH, LPO, LHA, and TU provided more inputs to GABAergic neurons than those to glutamatergic neurons. In the midbrain (Figure 4B), we found that MRN, SCm, and PAG provided the largest inputs to glutamatergic and GABAergic neurons in VTA and SNc. Unexpectedly, SCm provided a greater share of input to SNc glutamatergic neurons compared to other groups. In the hindbrain (Figure 4C), glutamatergic and GABAergic neurons in the VTA and SNc received a very similar proportion of input from the same brain regions. Compared to other areas, fewer input neurons were found in the cortex (Figure 4D). However, there were significant differences in the distribution of cortical inputs to glutamatergic and GABAergic neurons in the VTA and SNc. Compared to VTA and SNc GABAergic neurons, glutamatergic neurons received more inputs from MO, SS, ACA, RSP, and HPF. As the main input regions of the basal ganglia, striatum and pallidum were two very crucial upstream brain regions of the VTA and SNc. Indeed, compared to other brain regions, CP and ACB provided the largest proportion of input to glutamatergic and GABAergic neurons in the VTA and SNc (Figure 4E). In pallidum, VTA glutamatergic and GABAergic neurons received a higher proportion of input from SI, MA, and NDB than those in the SNc (Figure 4E).

### Whole-Brain Outputs of Glutamatergic and GABAergic Neurons in VTA and SNc

To verify whether glutamatergic and GABAergic neurons in VTA and SNc form reciprocal connections with their input nuclei, we also mapped their whole-brain outputs using Cre-dependent AAV-DIO-EYFP. Some representative coronal images were shown to illustrate the overall output patterns of glutamatergic and GABAergic neurons in the VTA and SNc (Supplementary Figure 4). Glutamatergic and GABAergic neurons in the VTA and SNc delivered outputs to discrete brain areas, including striatum (ACB, OT, LSr, and CP), pallidum (SI and NDB), hypothalamus (MPO, LPO, LHA, DMH, ZI, VMH, TU, and PH), midbrain (VTA, SNr, RR, SNc, DR, and PPN), and pons (CS, PRNr, and POR).

**FIGURE 5:**
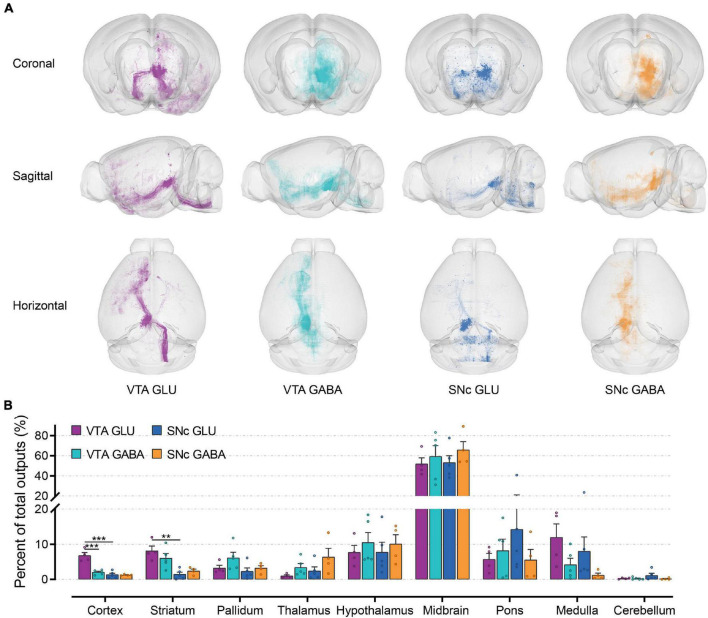
Whole-brain outputs of glutamatergic and GABAergic neurons in VTA and SNc. **(A)** 3D visualization of whole-brain output projections of the glutamatergic and GABAergic neurons in VTA and SNc from different view. **(B)** Proportions of output projections from nine brain areas. Mean ± SEM (*n* = 5 mice each for the VTA GABA and SNc GLU groups, *n* = 4 mice for the VTA GLU and SNc GABA). ***p < 0.001 and **p < 0.01. Only significant differences between the same cell type in different brain areas or between cell types in the same brain areas are marked; one-way ANOVA with Bonferroni correction. The percentages of outputs in nine brain areas are listed in Supplementary Data 2.

To observe the output distribution of glutamatergic and GABAergic neurons in the VTA and SNc more intuitively, we calculated the axon signal density matrix in the whole brain by 10 μm × 10 μm × 10 μm size voxels as the calculation unit. Then we visualized the density matrix as images and made the 3D rendering show (Figure 5A). After the injection site pixels (identified by the existence of labeled cell bodies) were excluded, the output projection to each brain region was quantified by the number of pixels occupied by the detected fluorescence fiber signals. To correct for the variability among different samples, the number of the pixels within each brain region was normalized by the total number of pixels containing fluorescence fiber signals pixels throughout the whole-brain. Just like the quantitative study of the input tracing, we first compared the output in nine grouped brain areas (Figure 5B). The largest proportion of output projections was found in the midbrain. Meanwhile, very few output projections were detected in the cerebellum. Compared to the other brain areas, we found that cortex, striatum, and medulla received more projections from VTA glutamatergic neurons.

### Quantitative Analysis of the Proportions of Whole-Brain Outputs to Glutamatergic and GABAergic Neurons in VTA and SNc

**FIGURE 6:**
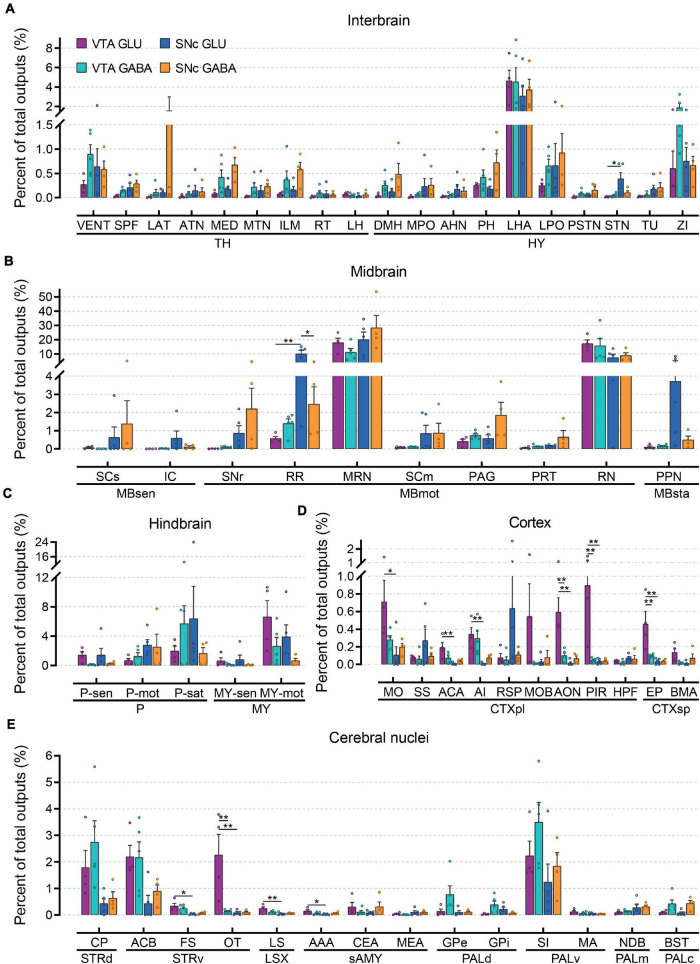
Quantitative analysis of whole-brain output projections to glutamatergic and GABAergic neurons in VTA and SNc. **(A)** Proportions of total outputs in interbrain region. **(B)** Proportions of total outputs in midbrain region. **(C)** Proportions of total outputs in hindbrain region. **(D)** Proportions of total outputs in cortex region. **(E)** Proportions of total outputs in cerebral nuclei region. Mean ± SEM (*n* = 5 mice each for the VTA GABA and SNc GLU groups, *n* = 4 mice each for the VTA GLU and SNc GABA groups). **p < 0.01 and *p < 0.05. Only significant differences between the same cell type in different brain regions or between cell types in the same brain regions are marked; one-way ANOVA with Bonferroni correction. Abbreviations of the 61 brain regions and their percentages of outputs are listed in Supplementary Table 1 **and** Data 2.

To further compare the whole-brain output projections of glutamatergic and GABAergic neurons in the VTA and SNc, we compared the proportions of outputs in 61 individual brain nuclei (Figure 6 and Supplementary Figure 5). Overall, compared to input distributions, the output distributions showed striking differences between cell types in the VTA and SNc.

In interbrain (Figure 6A), LHA received the heaviest projections from glutamatergic and GABAergic neurons in the VTA and SNc. In combination with the results of the input tracing, we found that LHA not only provided extensive inputs to glutamatergic and GABAergic neurons in the VTA and SNc but also acted as a major recipient from glutamatergic and GABAergic neurons in the VTA and SNc. Compared to other groups, the projection from SNc GABAergic neurons was much stronger in LAT, MED, ILM, DMH, PH, and LPO (Figure 6A). In addition, VENT and ZI received a higher proportion output from VTA GABAergic neurons compared to those from VTA glutamatergic neurons (Figure 6A). When examining the output projections in the midbrain (Figure 6B), we found that MRN and RN received the largest outputs from glutamatergic and GABAergic neurons in the VTA and SNc. Unlike the input distributions, the SCm and PAG did not received a higher proportion of output from glutamatergic and GABAergic neurons in the VTA and SNc (Figure 6B), indicating an asymmetric connectivity between SC/PAG and VTA/SNc. In the hindbrain (Figure 6C), glutamatergic and GABAergic neurons in the VTA and SNc delivered prominent different outputs to the same brain regions. Compared to GABAergic neurons, MY-mot received a higher proportion of output from glutamatergic neurons in the VTA and SNc. Compared to other areas, cortex received less output projections from glutamatergic and GABAergic neurons in the VTA and SNc (Figure 6D). However, some significant differences in the projection proportion in different subregions of cortex were also observed. Compared to other groups, VTA glutamatergic neurons provided more outputs to MO, ACA, AI, MOB, AON, PIR, EP, and BMA. In the striatum, CP and ACB received a greater proportion of output from glutamatergic and GABAergic neurons in the VTA and SNc (Figure 6E). In the pallidum, VTA GABAergic neurons delivered a higher proportion output to GPe, GPi, and SI (Figure 6E). These results showed that input and output nuclei to glutamatergic and GABAergic neurons in VTA and SNc were largely overlapping brain regions (Figures 4, 6). However, the connectivity strengths between the input and output nuclei were different (Figures 4, 6).

Using advanced viral tracing techniques combined with high-throughput three-dimensional imaging system, we systematically mapped and compared the whole-brain output connectivity of glutamatergic and GABAergic neurons in the VTA and SNc. However, using adeno-associated virus to trace the whole-brain output projections of glutamatergic and GABAergic neurons in VTA and SNc alone cannot determine whether the axonal fibers originating from the injection site are projected through or to the targeted brain region. Therefore, the results of our given output circuits can only provide a reference and inference for the study of output circuits in the VTA and SNc.

### Comparison Between Inputs and Outputs to Glutamatergic and GABAergic Neurons in the VTA and SNc

To further compare the inputs and outputs of glutamatergic and GABAergic neurons in VTA and SNc, we calculated the correlation coefficient (CC) between cell types in the VTA and SNc.

**FIGURE 7:**
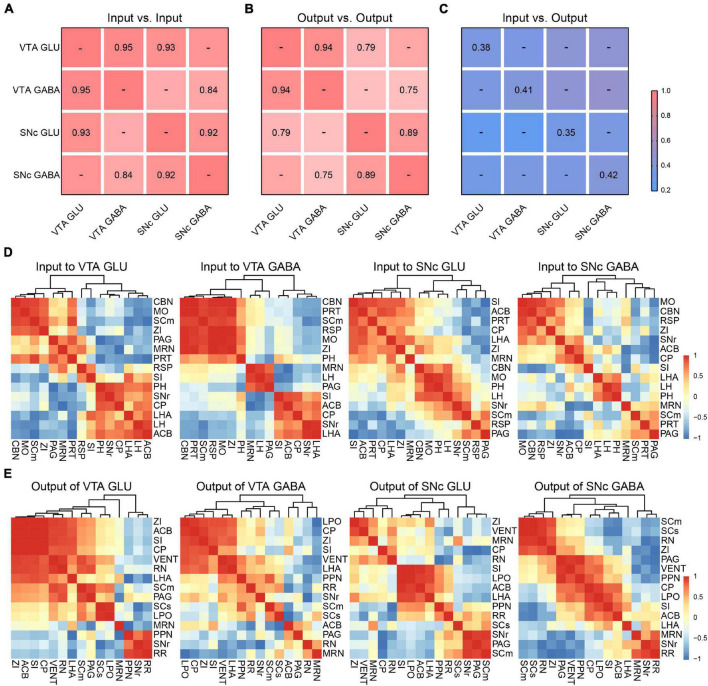
Comparisons of input and output distributions. **(A)** Matrix of correlation coefficients (CCs) between input distributions of each pair of cell types. CCs were computed at the spatial scale of the 67 major brain regions (Supplementary Data 1). **(B)** CCs between output distributions of each pair of cell types. CCs were computed at the spatial scale of the 61 major brain regions (Supplementary Data 2). **(C)** CCs between input and output distributions. CCs were computed at the spatial scale of the 54 major brain regions (Supplementary Data 2). **(D)** Patterns of Pearson’s correlation coefficient values and cluster trees showing the dissimilarities for the inputs of glutamatergic and GABAergic neurons in VTA and SNc. **(E)** Patterns of Pearson’s correlation coefficient values and cluster trees showing the dissimilarities for the outputs of glutamatergic and GABAergic neurons in VTA and SNc.

For input distribution, the CCs between all groups were high (Figure 7A), consistent with the results we observed earlier (Figures 2, 4). All these results indicated that the input patterns to the glutamatergic and GABAergic neurons in VTA and SNc were similar. Meanwhile, we also hierarchically clustered the correlation coefficients of inputs proportions of glutamatergic and GABAergic neurons in the VTA and SNc in main individual brain regions (Figure 7D). CBN, PRT, SCm, RSP, MO, and ZI showed a very high correlation in the VTA GABA group. Similarly, CBN, MO, SCm, and ZI also formed cluster in the VTA GLU group. These results indicated the input patterns from these brain regions to VTA glutamatergic neurons and GABAergic neurons were similar. Another similar cluster containing ACB, CP, SI, LHA, and SNr was also observed in SNc GLU and GABA group. Despite the high similarity, some correlations in two groups showed striking differences. For example, in the VTA GABA group, the MRN and LH were positively correlated. However, they were negatively correlated in the VTA GLU group. These results indicated that MRN and LH showed distinct input patterns to the VTA glutamatergic neurons and GABAergic neurons. The distinct correlation patterns in the SNc glutamatergic neurons and GABAergic neurons were also observed. These results indicated that despite the overall similarity among the input patterns of different neurons, there were still some detailed differences among the input patterns of different neurons, indicating that there might be different neurons innervating glutamatergic and GABAergic neurons in the same brain region.

For output distribution, the CCs between cell types were lower than those for input distribution (Figure 7B). Similarly, we also performed clustering analysis of output proportions of glutamatergic and GABAergic neurons in the VTA and SNc in individual brain regions (Figure 7E). Among four groups, different clusters were formed. For example, PPN and RN were positively correlated in the VTA GLU group, but negatively correlated in the VTA GABA group (Figure 7E). These results indicated that the output patterns and the collateral projections of glutamatergic neurons and GABAergic neurons in the VTA and SNc were distinct.

Finally, we calculated the CCs between the input and output distributions of each group (Figure 7C). We found that the CCs of input-output were much lower than input-input and output-output, reflecting the facts that input-output system possessed bidirectional connections but lacked of strong reciprocal connections in some brain regions.

## Discussion

In this study, we systematically mapped and compared the whole-brain input and output connectivity of glutamatergic and GABAergic neurons in the VTA and SNc using advanced viral tracing techniques combined with high-throughput three-dimensional imaging system (Supplementary Figure 6). Our results revealed that the input and output of glutamatergic and GABAergic neurons in the VTA and SNc formed reciprocal connections in some overlapping brain regions, but they had different proportions. Meanwhile, the input distributions were similar among different cell types in the VTA and SNc. However, unlike the input distributions, the output distributions of glutamatergic and GABAergic neurons in the VTA and SNc showed prominent differences. Glutamatergic neurons in the VTA and SNc generally delivered outputs to forebrain and hindbrain. In contrast, GABAergic neurons mainly send their axons in the interbrain and midbrain. These results revealed the structural basis underlying the brain function of the VTA and SNc and shed light on the treatment of neurological disorders associated with VTA and SNc dysfunctions.

### Cell-Type-Specific Inputs to the Glutamatergic and GABAergic Neurons in VTA and SNc

Classical anterograde and retrograde tracing methods have revealed that major inputs to VTA arise from numerous brain areas including the medial prefrontal cortex, LH, DR, ACB, PALv, LHA, and LDT (Geisler et al., 2007; Yetnikoff et al., 2014). However, traditional tracing methods lack cell-type specificity and have no ability to identify the whole-brain monosynaptic input of cell-type-specific neuronal population. Therefore, we used the rabies virus-mediated transsynaptic retrograde tracing to map the whole-brain monosynaptic inputs to glutamatergic and GABAergic neurons in VTA and SNc. We found that glutamatergic and GABAergic neurons in the VTA and SNc receive extensive inputs from widespread brain areas across the whole brain, including cortical plate (MOp, MOs, SS, AI, RSP, and VIS), cortical subplate (BLA), striatum (ACB, CP), pallidum (BST), thalamus (LH), hypothalamus (MPO, LPO, TU, LHA, and ZI), midbrain (MRN, VTA, IF, SNr, NOT, PAG, SNc, SC, APN, IPN, RR, CLI, and DR), and hindbrain (PRNc, GRN, IO, and RPA). These results reflected the higher sensitivity of the rabies virus-mediated transsynaptic retrograde tracing method.

Using the rabies virus-mediated transsynaptic retrograde tracing, Faget et al. (2016) have also mapped the whole-brain inputs to each transmitter-defined VTA cell types. The overall pattern and proportion of the afferents were similar across our studies. For example, we all observed that glutamatergic neurons in the VTA and SNc received more cortical inputs (e.g., MO, SS, ACA, RSP, and HPF) compared to VTA and SNc GABAergic neurons. Specifically, in thalamus, VTA GABAergic neurons received significantly more inputs from LH. In a recent circuit-based study, it was demonstrated that the inputs to the VTA from LH played roles in motivated behaviors by influencing VTA neuronal activity (Lammel et al., 2012). Therefore, according to our anatomical results, it is very likely that neurons in the LH preferentially innervated GABAergic neurons in the VTA to drive feedforward inhibition onto other neuron types in the VTA to express the aversive emotion.

Compared to thalamus, hypothalamus provided a higher proportion of input to glutamatergic and GABAergic neurons in the VTA and SNc, especially for LHA and ZI. Recently, Barbano et al. (2020) also demonstrated that a single VTA glutamatergic neuron received multiple hypothalamic excitatory inputs using electrophysiology and behavioral studies. These findings indicated that VTA glutamatergic neurons were activated by and required for innate defensive responses and the information of threatening stimuli to VTA glutamatergic neurons was relayed by LHA-glutamatergic neurons. Thus, these results were consistent with our studies that glutamatergic neurons in the VTA received direct inputs from LHA. Besides, the LHA-VTA circuit has been extensively studied and has been involved in multiple functions such as sleep regulation, reward, and food intake (Barbano et al., 2016; Tung et al., 2016; Sharpe et al., 2017; Blacktop and Sorg, 2019). Previous study has indicated that food intake and reward tended to activate the GABAergic neurons in LHA that projected to VTA (Barbano et al., 2016). In our results, we found that neurons in LHA sent more inputs to GABAergic neurons in VTA. Thus, it is likely that GABAergic neurons in LHA that project to VTA inhibited the GABAergic neurons in VTA to disinhibit the dopaminergic neurons in VTA to mediate the reward expression. However, considering the cell type heterogeneity of LHA neurons, which types of neurons in LHA preferentially innervate GABAergic neurons in the VTA needs to be investigated in future studies.

As for input distributions of the SNc, we found that SNc glutamatergic neurons received more inputs from SCm. The SCm has been found to be involved in regulating defensive response and locomotion (Liang et al., 2015; Wei et al., 2015). Thus, the biased inputs indicated that the glutamatergic neurons in SNc might also play an important role in regulating defense and locomotion.

In short, we took advantage of the high-throughput three-dimensional imaging system and provided a more comprehensive analysis and comparison of monosynaptic input to glutamatergic and GABAergic neurons in the VTA and SNc.

### Cell-Type-Specific Outputs of the Glutamatergic and GABAergic Neurons in VTA and SNc

Previous studies have identified VTA GABAergic neuron projections to the ACB in rats and mice; to LH in mice (Brown et al., 2012; van Zessen et al., 2012; Stamatakis et al., 2013); and to PAG, DR, and PALv in rat (Mitrovic and Napier, 2002; Kirouac et al., 2004). VTA glutamatergic neurons have been revealed to project to ACB and prefrontal cortex (PFC) in rats and mice and to LH, amygdala, and PALv in mice (Yamaguchi et al., 2011; Gorelova et al., 2012; Hnasko et al., 2012). Using viral-vector-mediated cell-type-specific anterograde tracing and classical retrograde tracing, Taylor et al. (2014) identified more widespread projections to limbic structures from both VTA glutamatergic and GABAergic neurons. They found that VTA glutamatergic neurons project heavily to LH, PALv/BST, and MA/OT and VTA GABAergic neurons project primarily to limbic structures other than PFC and ACB, including the LPO, MA, BST, CEA, LH, PALv, and DR. In this study we provided more systematic and objective analysis of the whole brain projection of glutamatergic neurons and GABAergic neurons in the VTA and SNc with automatic unsupervised methods. Glutamatergic neurons and GABAergic neurons in the VTA and SNc sent massive projections to multiple brain regions. These brain areas included striatum (ACB, OT, LSr, and CP), pallidum (SI and NDB), hypothalamus (MPO, LPO, LHA, DMH, ZI, VMH, TU, and PH), midbrain (VTA, SNr, RR, SNc, DR, and PPN), and pons (CS, PRNr, and POR). Specially, LHA received the heaviest projections from glutamatergic and GABAergic neurons in the VTA and SNc. Unlike the input distributions, the SCm and PAG did not receive a higher proportion of output projection from glutamatergic and GABAergic neurons in the VTA and SNc. Specifically, comparison of the projections of the VTA and SNc glutamatergic and GABAergic neuron, we observed that the brain regions innervated by VTA and SNc also project back to the VTA and SNc.

In general, we employed advanced viral tracing techniques combined with the precise whole-brain imaging system to extend our understanding of output circuits to glutamatergic and GABAergic neurons in VTA and SNc.

### Limitations of the Study

Although we employed advanced viral tracing techniques to map and compare the whole-brain input and output connectivity of glutamatergic and GABAergic neurons in VTA and SNc. However, this study has several potential caveats.

First, rabies virus-mediated transsynaptic retrograde tracing has been widely applied to map neural connectivity among specific types of neurons in specific brain regions, but given the possible differences in viral tropism, only a fraction of inputs to starter cells can be labeled and the number of labeled input neurons does not truly reflect the functional connectivity strength.

Second, although we revealed the input and output neural connectivity of different cell types in the VTA and SNc, it should be noted that the quantification of inputs and outputs depends largely on the location of the virus injection site and the spread of the virus. To avoid the risk as possible as we could, we checked whether the injection site of each sample was correct according to the starter cell location and the PI-stained cytoarchitecture information, even so it was hard to guarantee that the injection sites were completely accurate. However, we found that the majority of starter cells or cell bodies was located in the injection region and only few starter cells or cell bodies were outside the injection region. Thus, we inferred that this injection leakage has little impact on quantitative analysis.

Third, accumulating evidence indicates that VTA has combinatorial neurons that co-release glutamate and GABA (Root et al., 2014, 2018). We know that the output distributions of glutamatergic and GABAergic neurons in the VTA and SNc showed prominent differences, which indicated the proportion of neurons co-expressed glutamate and GABA would not be too high. But these co-expressed neurons may have some impacts the conclusion of this paper. Thus, future studies are expected to combine more advanced genetic and viral approaches to target diverse cell subtypes in the VTA and SNc.

In conclusion, we provided a comprehensive atlas of the input and output connectivity of glutamatergic and GABAergic neurons in VTA and SNc. This study systematically analyzed and compared inputs and outputs to specific cell types in the VTA and SNc. Our results reveal that the input patterns of different cell types in the VTA and SNc are highly similar, but the output projections show significant differences. These similarities and differences observed in our study may help to further our understanding of neural connectivity and provide new insight into the diverse functions of VTA and SNc.

## Data Availability Statement

The original contributions presented in the study are included in the article/Supplementary Material, further inquiries can be directed to the corresponding author/s.

## Ethics Statement

The animal study was reviewed and approved by the Animal Ethics Committee of Huazhong University of Science and Technology.

## Author Contributions

HG and QL conceived and designed the study. SA and ZF performed the experiments and analyzed the data. XLi and PZ performed the tracing experiments and embedded the samples. LD, ZD, and YH contributed to acquire the whole-brain dataset. SA, YL, XLiu, ZF, and AL participated in image processing and visualization. SA and HG drew the figures. SA, ZF, and XLi wrote the manuscript. All authors contributed to the article and approved the submitted version.

## Conflict of Interest

The authors declare that the research was conducted in the absence of any commercial or financial relationships that could be construed as a potential conflict of interest.

## Publisher’s Note

All claims expressed in this article are solely those of the authors and do not necessarily represent those of their affiliated organizations, or those of the publisher, the editors and the reviewers. Any product that may be evaluated in this article, or claim that may be made by its manufacturer, is not guaranteed or endorsed by the publisher.
