# Supplementary material for: A Whole-Brain Connectivity Map of VTA and SNc Glutamatergic and GABAergic Neurons in Mice
Source: Front Neuroanat. 2021 Dec 23;15:818242. doi: 10.3389/fnana.2021.818242 (PMC8733212; doi:10.3389/fnana.2021.818242)
Supplement: Supplementary file 1 [file Data_Sheet_1.docx]

Supplementary Material

# Supplementary Data

**Supplementary Data 1**. Quantification and comparison of whole-brain inputs of glutamatergic and GABAergic neurons in VTA and SNc.

**Supplementary Data 2**. Quantification and comparison of whole-brain outputs of glutamatergic and GABAergic neurons in VTA and SNc.

# Supplementary Figures and Tables

## Supplementary Figures

**
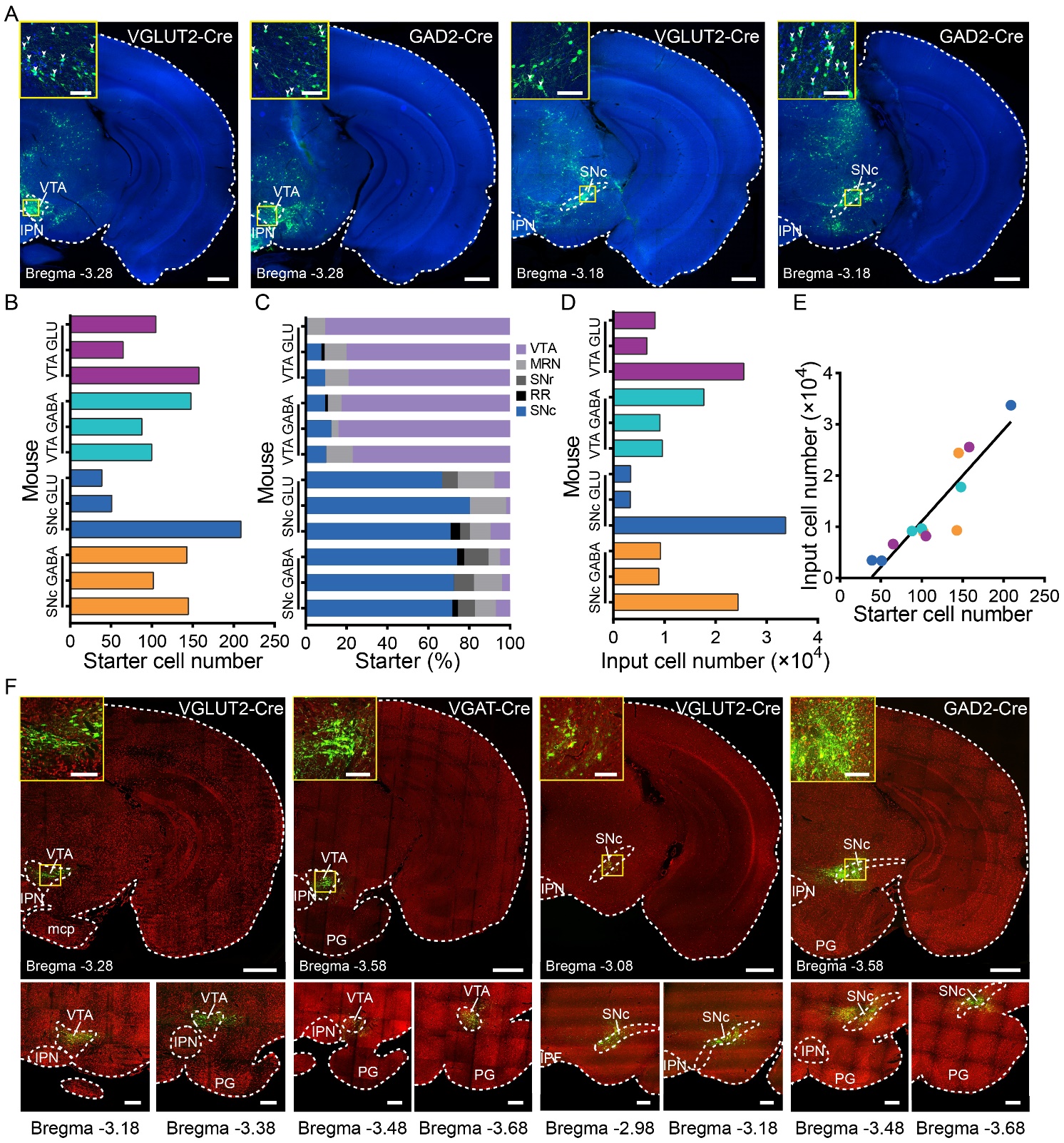
**

**Supplementary Figure 1. Demonstrating the specificity and accuracy of input and output tracing** **strategy.** (A) Representative coronal images near the injection sites for input tracing. Scale bar, 500 μm. Enlarged image of boxed area showing the starter cells (cyan, indicated by white arrowheads) and local input cells (green). Scale bar, 100 μm. (B) Numbers of starter cells in the individual animals. Purple, cyan, blue and orange represent VTA GLU, VTA GABA, SNc GLU and SNc GABA groups, respectively. (C) Proportions of labeled starter cells in injection regions. MRN, Midbrain reticular nucleus; SNr, Substantia nigra, reticular part; RR, Midbrain reticular nucleus, retrorubral area. (D) Numbers of transsynaptically labeled neurons (“input neurons”), each row represents the input neurons in each mouse. (E) A linear relationship between the number of starter cells and input cells. (F) Representative coronal images near the injection sites for output tracing. Scale bar, 500 μm. Enlarged image of boxed area showing the soma morphology of cells near the injection site. Scale bar, 100 μm. Bottom panel, representative coronal images near the injection sites. Scale bar, 250 μm.


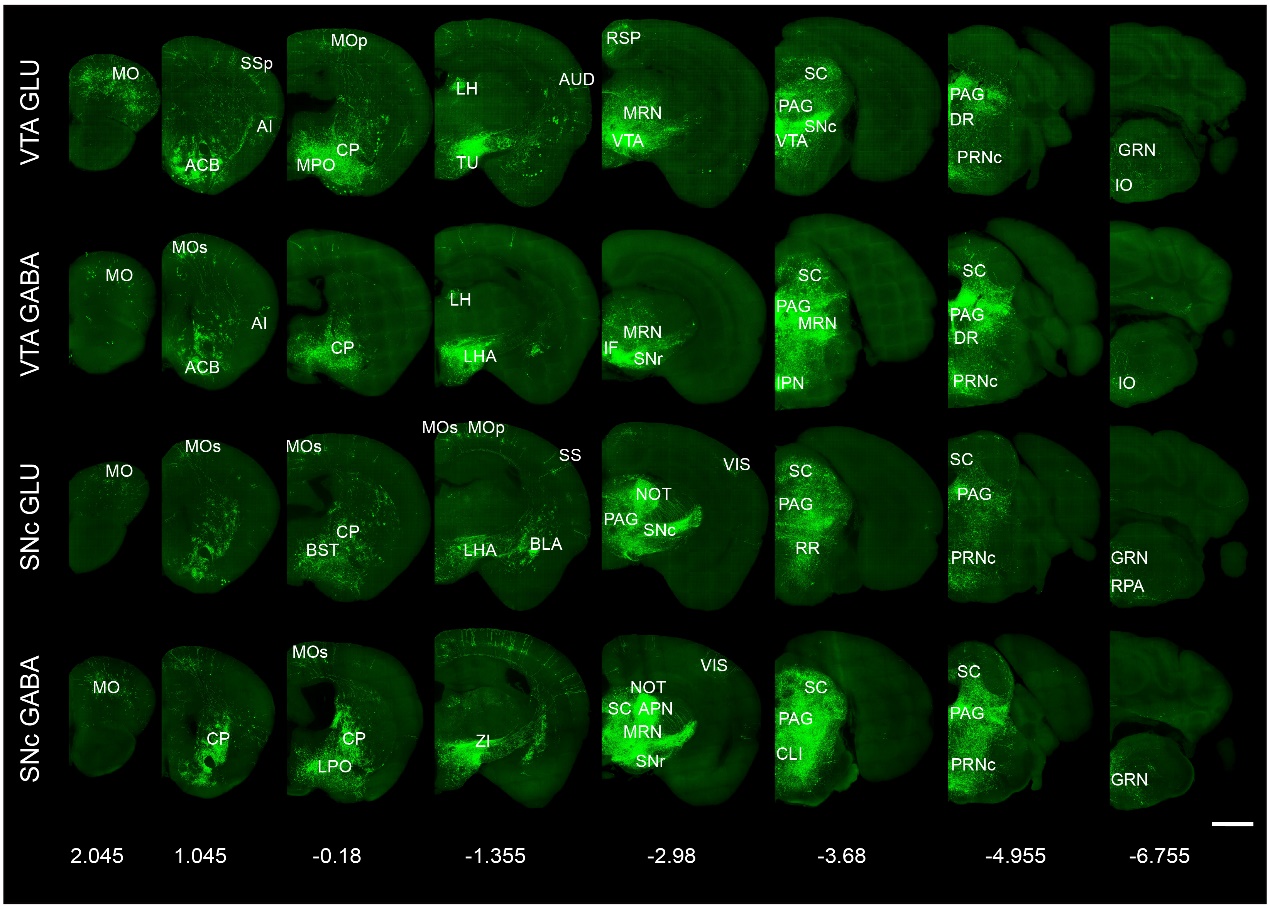


**Supplementary Figure 2. Overview of whole-brain monosynaptic inputs to glutamatergic and GABAergic neurons in VTA and SNc.** Representative coronal images showing labelling of monosynaptic inputs to glutamatergic and GABAergic neurons in VTA and SNc. Only the side ipsilateral to the injection site is shown. Scale bar, 1 mm.


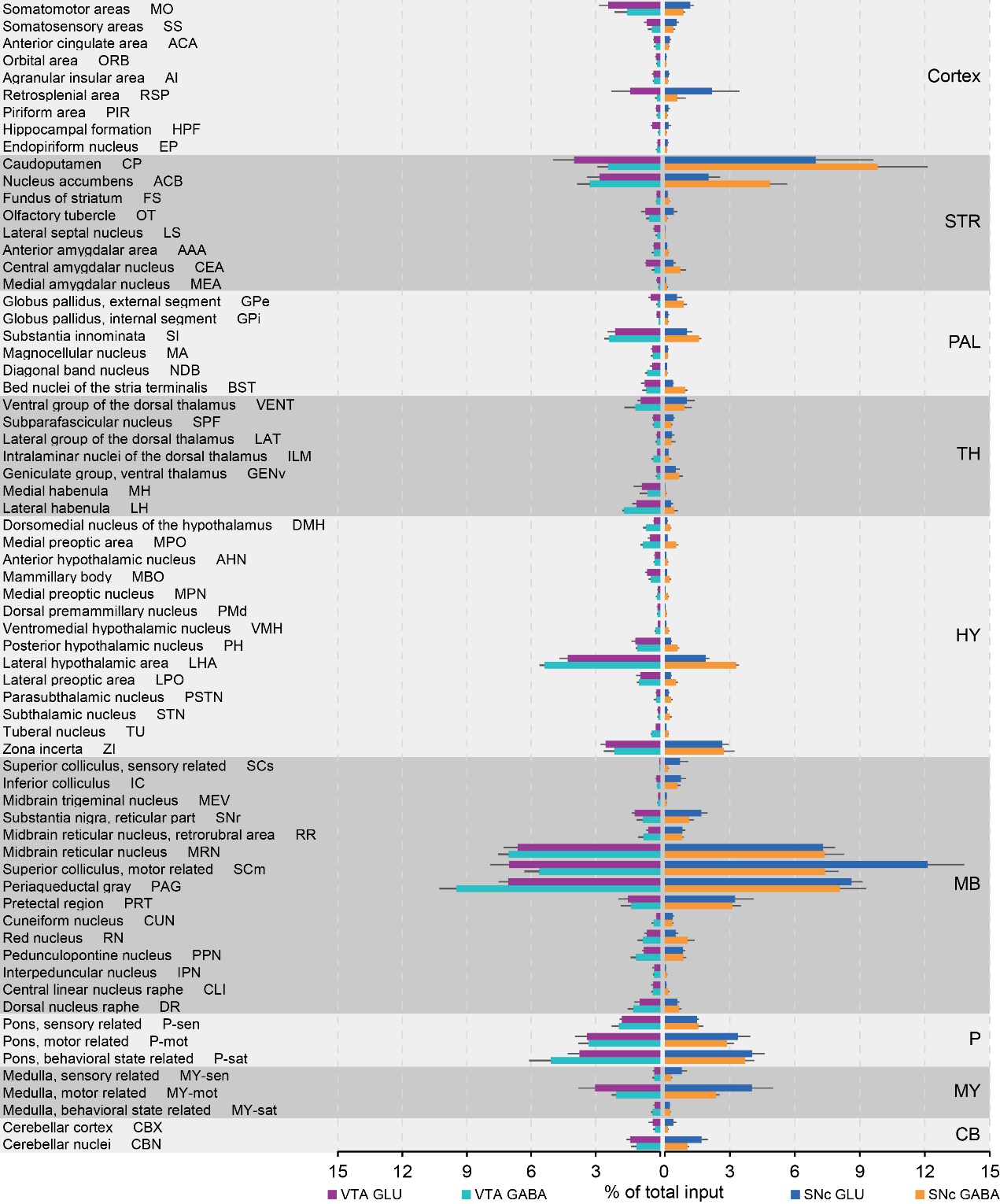


**Supplementary Figure 3. Whole-brain distributions of inputs to glutamatergic and GABAergic neurons in VTA and SNc**. Percentages of monosynaptic inputs to glutamatergic and GABAergic neurons in VTA and SNc in 67 brain areas. Mean ± SEM (n = 5 mice each for the VTA GLU, VTA GABA, SNc GLU and SNc GABA groups). Brain areas are divided into nine major brain structures. See **Supplementary Data 1** for detailed counts.


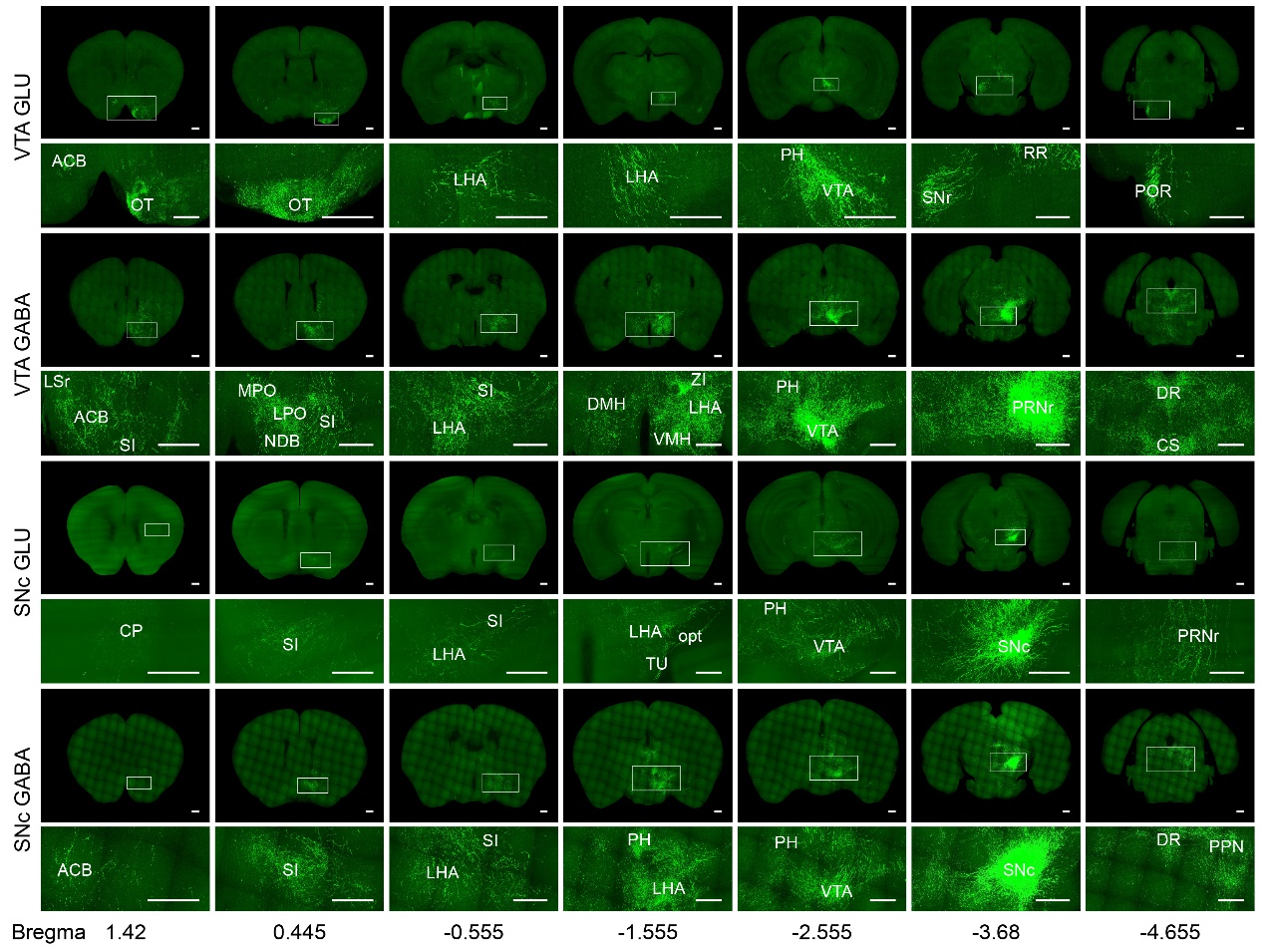


**Supplementary Figure 4. Overview of whole-brain output patterns of the glutamatergic and GABAergic neurons in VTA and SNc**. Representative coronal images showing output projections of glutamatergic and GABAergic neurons in VTA and SNc. Scale bar, 500 μm.


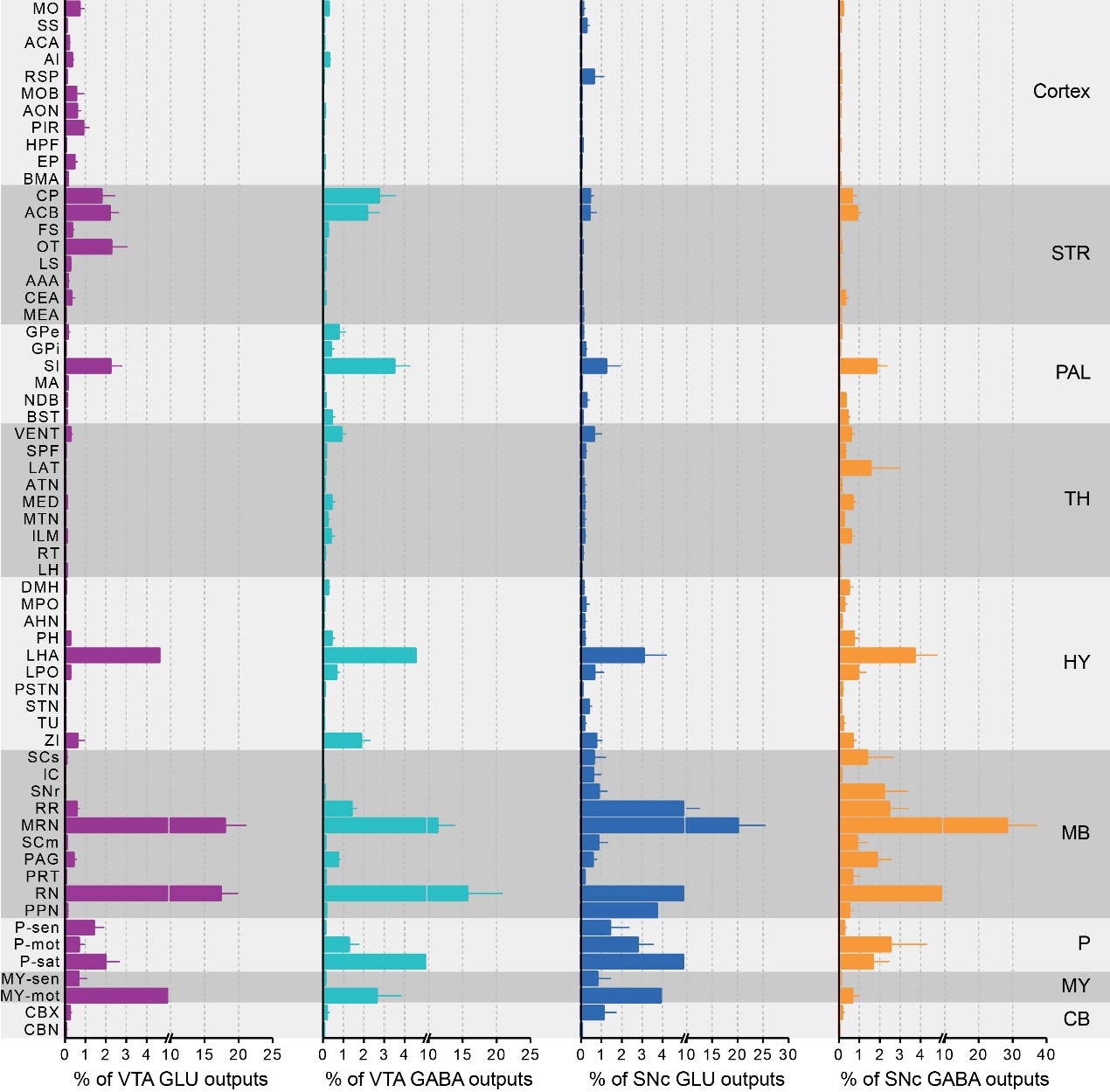


**Supplementary Figure 5. Whole-brain output projections to glutamatergic and GABAergic neurons in VTA and SNc**. Percentages of output projections to glutamatergic and GABAergic neurons in VTA and SNc in 61 brain areas. Mean ± SEM (n = 5 mice each for the VTA GABA and SNc GLU groups, n = 4 mice each for the VTA GLU and SNc GABA groups). Brain areas are divided into nine major brain structures. Abbreviations of the 61 brain regions and their percentages of outputs are listed in **Supplementary Table 1** and **Supplementary Data 2**.

**
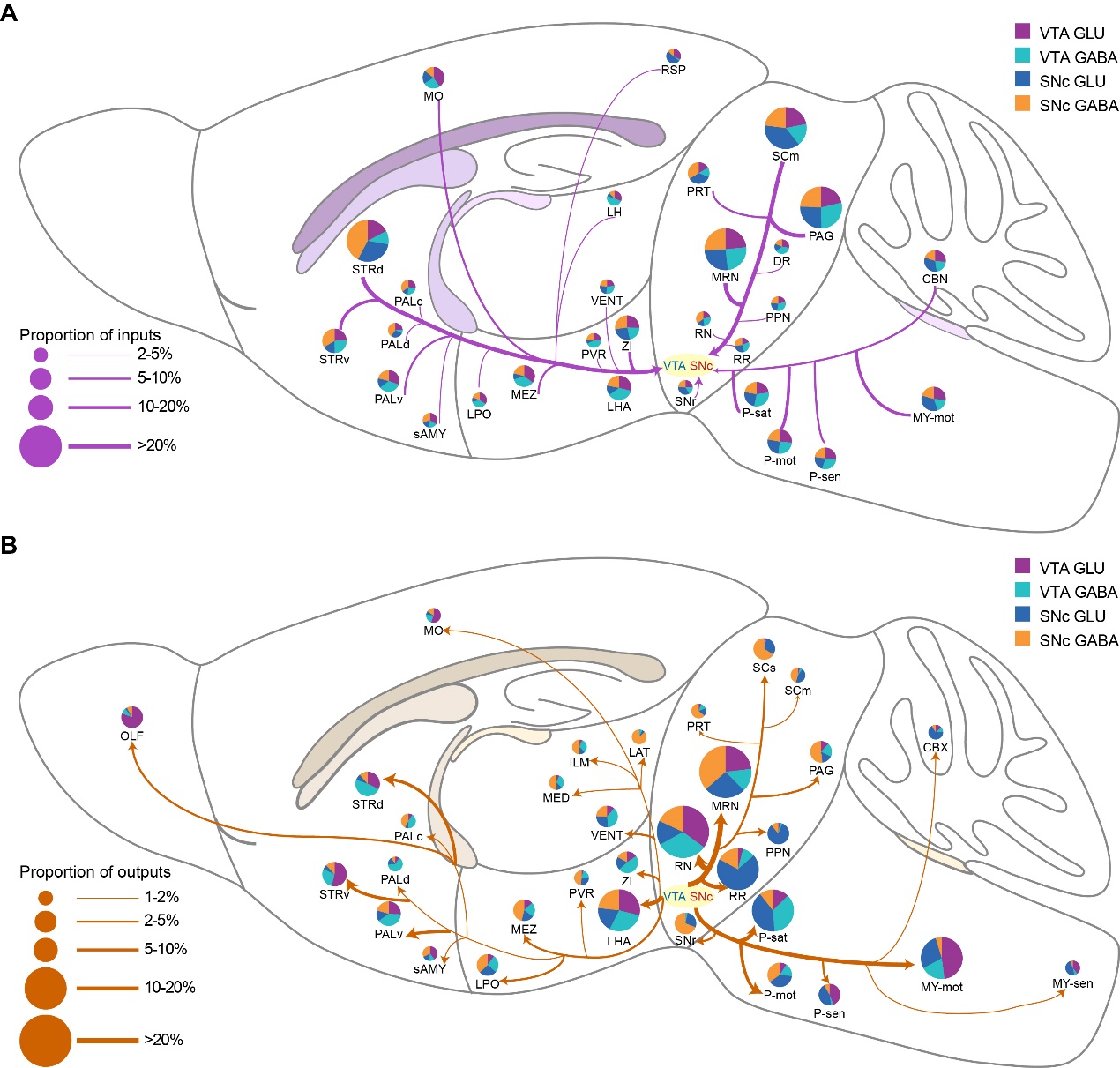
**

**Supplementary Figure 6. Whole-brain schematic of the input and output distributions** **to glutamatergic and GABAergic neurons in VTA and SNc.** (A) Whole-brain schematic of the inputs to glutamatergic and GABAergic neurons in VTA and SNc. (B) Whole-brain schematic of the outputs to glutamatergic and GABAergic neurons in VTA and SNc. Line thickness and the size of the pie charts represents the sum of the percentages. The details of abbreviations for brain regions are listed in **Supplementary Table 1**.

## Supplementary Table

**Supplementary Table 1 Abbreviation for brain areas and regions**

| **Areas** | | **Abbreviation for brain area** | |
| --- | --- | --- | --- |
| Cortex | Cortex | ACA | Anterior cingulate area |
|  | Cortex | AI | Agranular insular area |
|  | Cortex | AON | Anterior olfactory nucleus |
|  | Cortex | BLA | Basolateral amygdalar nucleus |
|  | Cortex | BMA | Basomedial amygdalar nucleus |
|  | Cortex | EP | Endopiriform nucleus |
|  | Cortex | HPF | Hippocampal formation |
|  | Cortex | MO | Somatomotor areas |
|  | Cortex | MOB | Main olfactory bulb |
|  | Cortex | MOp | Primary motor area |
|  | Cortex | MOs | Secondary motor area |
|  | Cortex | OLF | Olfactory areas |
|  | Cortex | ORB | Orbital area |
|  | Cortex | PIR | Piriform area |
|  | Cortex | RSP | Retrosplenial area |
|  | Cortex | SS | Somatosensory areas |
|  | Cortex | VIS | Visual areas |
| STR | Striatum | AAA | Anterior amygdalar area |
|  | Striatum | ACB | Nucleus accumbens |
|  | Striatum | CEA | Central amygdalar nucleus |
|  | Striatum | CP | Caudoputamen |
|  | Striatum | FS | Fundus of striatum |
|  | Striatum | LS | Lateral septal nucleus |
|  | Striatum | LSr | Lateral septal nucleus, rostral part |
|  | Striatum | MEA | Medial amygdalar nucleus |
|  | Striatum | OT | Olfactory tubercle |
|  | Striatum | sAMY | Striatum-like amygdalar nuclei |
|  | Striatum | STRd | Striatum dorsal region |
|  | Striatum | STRv | Striatum ventral region |
| PAL | Pallidum | BST | Bed nuclei of the stria terminalis |
|  | Pallidum | GPe | Globus pallidus, external segment |
|  | Pallidum | GPi | Globus pallidus, internal segment |
|  | Pallidum | MA | Magnocellular nucleus |
|  | Pallidum | NDB | Diagonal band nucleus |
|  | Pallidum | PALc | Pallidum, caudal region |
|  | Pallidum | PALd | Pallidum, dorsal region |
|  | Pallidum | PALv | Pallidum, ventral region |
|  | Pallidum | SI | Substantia innominata |
| TH | Thalamus | ATN | Anterior group of the dorsal thalamus |
|  | Thalamus | ATN | Geniculate group, ventral thalamus |
|  | Thalamus | ILM | Intralaminar nuclei of the dorsal thalamus |
|  | Thalamus | LAT | Lateral group of the dorsal thalamus |
|  | Thalamus | LH | Lateral habenula |
|  | Thalamus | MED | Medial group of the dorsal thalamus |
|  | Thalamus | MH | Medial habenula |
|  | Thalamus | MTN | Midline group of the dorsal thalamus |
|  | Thalamus | RT | Reticular nucleus of the thalamus |
|  | Thalamus | SPF | Subparafascicular nucleus |
|  | Thalamus | VENT | Ventral group of the dorsal thalamus |
| HY | Hypothalamus | AHN | Anterior hypothalamic nucleus |
|  | Hypothalamus | DMH | Dorsomedial nucleus of the hypothalamus |
|  | Hypothalamus | LHA | Lateral hypothalamic area |
|  | Hypothalamus | LPO | Lateral preoptic area |
|  | Hypothalamus | MBO | Mammillary body |
|  | Hypothalamus | MEZ | Hypothalamic medial zone |
|  | Hypothalamus | MPN | Medial preoptic nucleus |
|  | Hypothalamus | MPO | Medial preoptic area |
|  | Hypothalamus | PH | Posterior hypothalamic nucleus |
|  | Hypothalamus | PMd | Dorsal premammillary nucleus |
|  | Hypothalamus | PSTN | Parasubthalamic nucleus |
|  | Hypothalamus | PVR | Periventricular region |
|  | Hypothalamus | STN | Subthalamic nucleus |
|  | Hypothalamus | TU | Tuberal nucleus |
|  | Hypothalamus | VMH | Ventromedial hypothalamic nucleus |
|  | Hypothalamus | ZI | Zona incerta |
| MB | Midbrain | APN | Anterior pretectal nucleus |
|  | Midbrain | CLI | Central linear nucleus raphe |
|  | Midbrain | CUN | Cuneiform nucleus |
|  | Midbrain | DR | Dorsal nucleus raphe |
|  | Midbrain | IC | Inferior colliculus |
|  | Midbrain | IF | Interfascicular nucleus raphe |
|  | Midbrain | IPN | Interpeduncular nucleus |
|  | Midbrain | MEV | Midbrain trigeminal nucleus |
|  | Midbrain | MRN | Midbrain reticular nucleus |
|  | Midbrain | NOT | Nucleus of the optic tract |
|  | Midbrain | PAG | Periaqueductal gray |
|  | Midbrain | PPN | Pedunculopontine nucleus |
|  | Midbrain | PRT | Pretectal region |
|  | Midbrain | RN | Red nucleus |
|  | Midbrain | RR | Midbrain reticular nucleus, retrorubral area |
|  | Midbrain | SC | Superior colliculus |
|  | Midbrain | SCm | Superior colliculus, motor related |
|  | Midbrain | SCs | Superior colliculus, sensory related |
|  | Midbrain | SNc | Substantia nigra, compact part |
|  | Midbrain | SNr | Substantia nigra, reticular part |
|  | Midbrain | VTA | Ventral tegmental area |
| P | Pons | CS | Superior central nucleus raphe |
|  | Pons | P-mot | Pons, motor related |
|  | Pons | POR | Superior olivary complex, periolivary region |
|  | Pons | PRNc | Pontine reticular nucleus, caudal part |
|  | Pons | PRNr | Pontine reticular nucleus |
|  | Pons | P-sat | Pons, behavioral state related |
|  | Pons | P-sen | Pons, sensory related |
| MY | Medulla | GRN | Gigantocellular reticular nucleus |
|  | Medulla | IO | Inferior olivary complex |
|  | Medulla | MY-mot | Medulla, motor related |
|  | Medulla | MY-sat | Medulla, behavioral state related |
|  | Medulla | MY-sen | Medulla, sensory related |
|  | Medulla | RPA | Nucleus raphe pallidus |
| CB | Cerebellum | CBN | Cerebellar nuclei |
|  | Cerebellum | CBX | Cerebellar cortex |
